# Nursing Professionalism: A Scoping Review of Implementation Level, Evaluation Instruments, Influential Factors, and Intervention Strategies

**DOI:** 10.1155/2024/7272296

**Published:** 2024-08-20

**Authors:** Jing Jiang, Mengyuan Liu, Yinglan Li, Hongmei Gao, Lingyun Tian

**Affiliations:** ^1^ Xiangya School of Nursing Central South University, Changsha, China; ^2^ Teaching and Research Section of Clinical Nursing National Clinical Research Center of Geriatric Disorder Xiangya Hospital of Central South University, Changsha, China; ^3^ Department of Nursing the First Affiliated Hospital of USTC Division of Life Sciences and Medicine University of Science and Technology of China, Hefei, China

## Abstract

**Aim:**

The objective of this study is to provide a comprehensive analysis and synthesis of pertinent research on the subject of nursing professionalism.

**Background:**

The number of studies documenting nursing professionalism has been consistently increasing over the years; however, a comprehensive synthesis and analysis of the evidence presented in these reports is currently lacking. *Evaluation*. The scope reviews were conducted using electronic databases including PubMed, Web of Science, Cochrane Library, Embase, Chinese National Knowledge Infrastructure (CNKI), Chinese Science and Technology Periodical Database (VIP), and Wanfang database. *Key Issues*. This review included 15 studies and identified 4 significant pertinent issues: (1) the need for further investigation into the implementation level of nursing professionalism; (2) the development of culturally sensitive instruments to assess nursing professionalism; (3) an in-depth exploration of the associated factors influencing nursing professionalism; and (4) the critical implementation of diverse intervention strategies to enhance nursing professionalism.

**Conclusions:**

This review presents a comprehensive overview of the implementation level, assessment tools, influential factors, and intervention strategies in nursing professionalism research. In addition, it emphasizes the future research direction in this field. *Implications for Nursing Management.* Nursing administrators need to understand the significance of improving the professional education and training of nurses, fostering a conducive work environment, and providing support for their active development of professional skills and nursing professionalism. These factors collectively contribute to the long-term professional development of nurses and enhance the quality of patient care.

## 1. Introduction

Nursing professionalism serves as a guiding principle for nurses, ensuring patient safety and the delivery of high-quality nursing care. It underscores the significance of societal service, deeply rooted in the essence of humanity, thereby conferring a distinct meaning to nursing professionalism. Jiang et al. [1] believed that nursing professionalism encompasses the integration of fundamental practical concepts, value orientation, professional personality, professional standards, and professional style exhibited by nurses in their interactions with patients, which is a reflection of the nurses' attitudes and behaviors toward nursing work. Hafferty [2] concluded that professional competence, self-regulation, honor and integrity, altruism, respect, teamwork, responsibility, and continuous learning constitute the fundamental components of nursing professionalism. Miller [3] employed the prevalent professional perspectives of sociologists and nursing managers to develop the model of the wheel of professionalism in nursing based on ethical principles articulated in the American Nurses Association's (ANA) Code of Ethics for nurses policy statement. In the context of education in a university setting and scientific background in nursing, the model defines the professional spirit from the behavioral perspective, which includes (1) continuing education and competency; (2) adherence to the code for nurses; (3) professional organization participation; (4) publication and communication; (5) self-regulation and autonomy; (6) community service orientation; (7) theory: development, use, and evaluation; and (8) research: development, use, and evaluation. The term *nursing professionalism* refers to the fundamental professional ideas, attitudes, and values that nurses demonstrate in their nursing practice [4, 5], which includes the service concept of “*patient-center*ed,” the professional attitude of “*patient interests above all*,” and the values of “*promoting the physical and mental health of patients*.”

Nursing professionalism plays an important guiding role in the nursing profession. It highlights ethical principles, beliefs, and life perspectives and also represents the moral development, civilization, and spiritual attitude of nurses [6]. Nursing professionalism exerts a positive impact on elevating nursing standards, enhancing job satisfaction, promoting the sustained choice of the nursing profession, and mitigating the turnover rate among the nursing staff [7–11]. The cultivation and perpetuation of nursing professionalism are imperative for the development of exceptional nurses. The establishment of a truly exemplary nursing profession necessitates comprehensive legislation, a compassionate social environment, and most importantly, collaborative efforts from healthcare practitioners, educators, and administrators [12].

Studies on the implementation level of the nursing professionalism in clinical practice have been extensively conducted in various countries and regions. However, there is currently a dearth of empirical evidence exploring the nursing professionalism instruments in various cultural contexts, the associated influential factors, and the diversified intervention strategies. In this review, the assessment tools, influential factors, and intervention strategies of nursing professionalism were systematically evaluated based on the reporting framework for scope review proposed by Arksey and O'Malley in 2005 [13]. This comprehensive assessment aims to provide a reference for future studies.

## 2. Methods

### 2.1. Refining the Research Inquiry

To ensure the smoothness of this scoping review, the research questions were developed as follows:

1. What is the implementation level of nursing professionalism in clinical practice?
2. What assessment tools are available for the evaluation of nursing professionalism?
3. What are the key factors that influence nursing professionalism?
4. What effective interventions can be implemented to enhance nursing professionalism?

### 2.2. Identifying Pertinent Studies

The literature for this review was obtained by conducting comprehensive searches in seven electronic databases, including PubMed, Web of Science, Cochrane Library, Embase, CNKI, VIP, and Wanfang. These databases were searched from their inception until 13 July 2023. A combination of subject terms and free words was used to ascertain the topic of all relevant research, and the Boolean operators *OR* and *AND* were utilized to amalgamate the outcomes. The search theme was as follows: (a) *Professional spirit*, *Professionalism medical professionalism*, *Professionalism education*, *Occupational spirit*, and *Vocational spirit* and (b) *Nurses*, *nursing*, *Nursing students*, *Nursing personnel*, *Registered nurses*, and *Pupil nurses*. The specific strategies for retrieving data from the database can be found in Supplemental File 1.

### 2.3. Selecting Relevant Studies

The studies included in this review met the following criteria: (a) they were written in English or Chinese; (b) the subjects of the study were nurses or nursing students; and (c) they included the implementation level of nursing professionalism, instruments for measuring nursing professionalism, associated influential factors, or intervention strategies. Studies were excluded if (a) full text was not accessible; (b) their results had been duplicated in another article; and (c) they belonged to categories such as reviews, general comments, editorials, or case reports.

**Figure 1:**
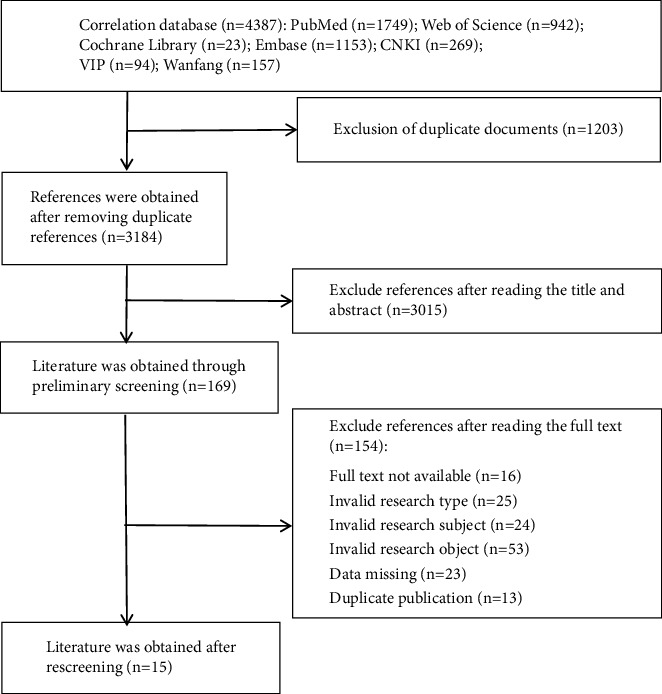
Flowchart of literature screening.

A total of 4,387 studies (Figure 1) were identified and imported into the NoteExpress software. After removing duplicates, 3,184 studies remained. These studies were further evaluated based on their titles and abstracts to determine their compliance with the inclusion and exclusion criteria. Subsequently, 3,015 studies that did not meet the inclusion criteria were excluded, and the remaining 169 full-text studies were retrieved and independently assessed by two co-first authors. Following a meticulous examination of the inclusion and exclusion criteria, 15 studies were finally included in this review.

### 2.4. Data Extraction

Two researchers independently extracted data using a designated data extraction form, under the close supervision of another researcher. The extracted information encompassed details such as authorship, publication year, country of origin, subjects, study design, implementation level of nursing professionalism, assessment tools, influential factors, intervention strategies, and intervention duration.

## 3. Results

### 3.1. Description of Studies

A total of 15 papers were published between 2014 and 2023. The majority of studies originated from China (*n* = 8), and the remaining papers were contributed by Japan (*n* = 3), Korea (*n* = 2), India, and Czechoslovakia (*n* = 1 each). The content of the study included the implementation level (*n* = 8), assessment tools (*n* = 14), the influential factors (*n* = 12), and the intervention strategies (*n* = 3). The study design encompassed a range of methodologies, including cross-sectional studies (*n* = 9), quasi-experimental studies (*n* = 1), randomized controlled trials (*n* = 2), qualitative studies (*n* = 1), and mixed studies (*n* = 2). The subjects consisted of both nurses (*n* = 10) and nursing students (*n* = 5).  Table 1 shows the details of the included studies.

### 3.2. Implementation Level of Nursing Professionalism Level

Among the 15 studies included, 8 papers discussed the implementation level of nursing professionalism in clinical practice in different regions. The existing research conducted in China indicates that undergraduate nursing students possess a moderate level of nursing professionalism [5, 22]. A separate study by Ma et al. [17] reveals a prevalent decline in the perception of nursing professionalism among low-income nurses. The nursing professionalism among the surveyed nurses from seven Chinese third-class A general hospital nurses was marginally lower than that of Korean-American nurses and similar to the average level in the United States [18]. Kurucová [20] conducted a survey on Czechoslovakian nurses with a moderate level of nursing professionalism, while Tanaka et al. [25] found a poor level of nursing professionalism among Japanese nurses.

### 3.3. Assessment Tools of Nursing Professionalism

Among the 15 publications, a total of 7 assessment tools were examined.  Table 2 shows detailed information of the assessment tools of nursing professionalism. Currently, the commonly used assessment tools have a number of limitations. The Chinese version of Hall's Professionalism Inventory (HPI) [22] exhibits robust reliability, effectiveness, and cultural sensitivity. However, stratified random sampling of various populations is still required to further corroborate the results of the confirmatory factor analysis and further refine the sampling technique used in the exploratory factor analysis of the scale. Based on a theoretical model, the Behavioral Inventory for Professionalism in Nursing (BIPN) (Miller, 1985) exhibits good reliability and validity. Nevertheless, its general applicability needs to be confirmed considering cultural variations. The Nurses' Professionalism Inventory (NPI) [24] is a reliable tool with a high degree of fit for each of its factors, while it lacks validity testing and does not adhere to any specific theoretical model during its development process, and the Korean-Nursing Profession Value (KNPV) [27] has the same problem. The self-designed questionnaire [17] has gathered concentrated expert consultation opinions on indicators at all levels. These experts possess high motivation and authority, ensuring the questionnaire's scientific rigor. However, it is imperative to further consider the completeness of index construction due to insufficient letters consulted with experts. The Nurse Professionalism Scale (NPS) (Gyung, 2022) demonstrates reliability and a rigorous design, but it suffers from a limited sample size and lacks a large multicenter survey. Despite demonstrating excellent internal consistency and stability, the nurse professionalism questionnaire [23] is limited in its research scope and requires careful consideration of its authority.

### 3.4. Influential Factors of Nursing Professionalism

Among the 15 publications, 10 articles were related to the influential factors of nursing professionalism, mainly including individual and organizational factors. Individual factors include gender, personal abilities, volunteer application, education level, and job position. In the existing studies, researchers have confirmed the effect of these influential factors on nursing professionalism. Specifically, male nurses tend to demonstrate comparatively lower levels of professionalism in comparison to their female counterparts [5, 18, 23]; the level of nursing professionalism is positively associated with critical thinking abilities, self-leadership skills, humanistic care capabilities, and self-efficacy [17, 21, 22, 27]; the level of nursing professionalism of nursing students is significantly elevated when they autonomously select the field [5, 18]; the higher level of education raises the standard for the level of nursing professionalism [5, 18, 23, 26]; in addition, nurses in higher job positions exhibit the higher level of nursing professionalism [20, 26]. Organizational factors such as rules and regulations, decision-making authority, and organizational culture also significantly affect nurses' professionalism [21].

### 3.5. Intervention Methods for Nursing Professionalism

Intervention studies on nursing professionalism were conducted in 3 out of the 13 articles [14–16]. Intervention methods mainly include narrative medicine education [14], health communication courses [15], and penguin style training [16]. Xue et al. [14] cultivated students' narrative ability through storytelling, careful reading, reflective writing, sharing, and discussions, and confirmed that narrative medicine can enhance students' nursing professionalism after a 12-month intervention study. In addition, the study reaffirmed the significance of empathy and humanistic care. Social media play a pivotal role in health communication courses, and Sun et al. [15] instructed nursing students on the effective utilization of social media platforms while monitoring their progress in developing social media competencies. After a 3-month intervention study, the social media capabilities of nursing students were significantly improved after receiving health communication education, and the utilization of social media platforms exerted a positive impact on the professionalism of nursing students. Dong and Shao [16] proposed the penguin style cultivation method for nursing professionalism. Specifically, a penguin style cultivation teaching group was first established, including nursing professional tutors, mental mentors, supervision tutors, and training partners, and then the penguin style training method was employed. The knowledge training of nurses was entrusted to professional tutors, while the attitude training was overseen by mental tutors. Skill training of nurses was facilitated by training partners, and supervision tutors were assigned the responsibility of overseeing teaching plans and methods, ultimately leading to a significant enhancement in nurses' professionalism.

## 4. Discussion

The importance of nursing professionalism lies in its promotion of patient health, maintenance of medical quality and safety, attention to holistic patient needs, the transmission of professional responsibility and values, and enhancement of nurses' career satisfaction. These attributes represent the fundamental qualities and attitudes that nurses must possess [30]. The concept of nursing professionalism should not be limited to a mere professional ethic, but rather as an ethics framework for nursing and even the broader healthcare industry. Therefore, it is essential to provide an overview of current research on nursing professionalism for nursing administrators and to maintain the stability of the nursing team.

In recent years, there has been a growing global interest in nursing professionalism, as evidenced by the findings of this study. Notably, Asian countries have shown particular attention to this aspect, which may be related to the high turnover rate of nurses in these regions [31, 32]. Consequently, research on nursing professionalism is seen as a means to enhance professional identity within the nursing profession.

The future trajectory of nursing professionalism warrants further investigation. Currently, disparities exist in research findings regarding the level of nursing professionalism across different countries and regions. For example, the level of professionalism among nurses in Czechoslovakia is considered moderate [20], whereas the professionalism level among nurses in Japan is comparatively low [25]. This disparity may be attributed to the absence of a unified connotation of nursing professionalism. Connotations are context-specific, and cultural and institutional contexts can influence the definition of connotations [33]. The connotation of nursing professionalism in China primarily focuses on the individual level, encompassing practice concepts, professional attitudes, value pursuits, professional ethics, and professional abilities. In other countries, the connotation of nursing professional spirit is directly implemented at the personal, interpersonal, and public levels [21]. The in-depth analysis of connotations and the clarification of variable indicators can guide further related research, and logical analysis methods should be applied to delineate specific issues [34]. Currently, inadequate breadth and depth of research along with ambiguous definitions of connotations have led to some studies deviating from objective facts. In the future, it is necessary to elucidate the connotations, structural components, and key points, clarify the ambiguous connotation, unify the relevant connotation, and further explore the level of professionalism of nursing. This review serves as a valuable reference for future research, the development of assessment scales, and the formulation of relevant intervention measures and policies in the field of nursing professionalism.

We urgently need to develop a culturally specific assessment tool for nursing professionalism that can be applied to diverse cultural backgrounds. Accurate measurement of nursing professionalism can assist nursing staff in forming more standardized and unified nursing behaviors [35]. The levels of nursing professionalism vary across different countries and regions, leading to different requirements for measurement instruments and challenges in the coordination of measurement standards and indicators. The limitations and imbalances of measurement instruments also restrict the comprehensiveness of assessment results, leading to one-sided and misleading evaluations [36]. Currently, the nursing professionalism assessment tool developed by Hall and Miler is grounded in a theoretical framework and extensively studies professionalism as a comprehensive concept, with broad applications [37–40]. Moreover, certain researchers focus on specific attributes of nursing professionalism and use corresponding scales as assessment tools [24]. At present, the development process of some research instruments lacks a specific theoretical model; some research instruments have not undergone reliability and validity testing, leading to limited research scope and a lack of multicenter and large-sample investigations. In summary, current assessment tools for nursing professionalism are still imperfect, as they lack clear evaluation effects, robust validity, and specificity. In the future, researchers should develop assessment tools with local characteristics based on the distinctive features of nursing professionalism in diverse regional cultural backgrounds. Furthermore, these tools should prioritize scientific rigor, reliability, and practicality while undergoing rigorous validation and refinement through relevant research studies.

The factors influencing nursing professionalism necessitate further exploration. The results of our scope review indicate that nursing professionalism is influenced by many complex factors. In terms of individual factors, studies mainly focus on gender, personal abilities, and career choice. Previous studies have demonstrated that male nurses tend to exhibit lower overall professionalism scores compared to their female counterparts [5, 18]. In addition, the level of nursing professionalism is positively associated with critical thinking abilities, self-leadership skills, and humanistic care capabilities. In China, due to the disproportionate number of candidates in college entrance examinations, colleges and universities are compelled to reassign unsuccessful candidates from their initial voluntary majors to undersubscribed majors. This process is commonly referred to as transfer. Nursing students who are transferred to the nursing profession have deep-seated psychological resistance to the nursing profession and experience strong emotional discrepancies due to the transfer results, which in turn affect their professionalism [17, 18]. In terms of organizational factors, the sense of responsibility and professionalism of nurse educators can be enhanced through adherence to rules and regulations, delegation of decision-making power to nurses by leaders, and fostering a positive organizational atmosphere [21]. These interventions will facilitate nurses in recognizing their intrinsic value, thereby fostering the cultivation of appropriate professional values. Nursing professionalism is an integral part of professional culture, necessitating comprehensive consideration of social and psychological factors and cultural traditions in future research. However, nursing professionalism is embedded within the professional culture, and future research should fully consider the influence of organizational managers, social psychological factors, and cultural traditional factors. The current research predominantly relies on cross-sectional surveys, while cross-sectional surveys are limited in their ability to elucidate intricate phenomena, establish causal relationships, and make future predictions. There are few reports on the interaction and causal mechanisms among various influential factors. Therefore, multicenter, large-sample, specific population, and longitudinal studies based on different cultural backgrounds are required to fully explore the factors affecting nursing professionalism.

Diversified interventions play a crucial role in enhancing nursing professionalism. The intervention study on nursing professionalism is still in its developmental stage, and its positive impact has been preliminarily validated, but there is still significant potential for further development and improvement. Currently, interventions aimed at enhancing nursing professionalism mainly include narrative medicine education [14], health communication courses [15], and penguin style training [16]. Specifically, narrative medicine education can be used to facilitate nurses' self-reflection, foster their empathetic abilities [41], and understand patients' real feelings from different perspectives, thus shaping the correct professional values. However, this study adopted voluntary participation and self-report surveys, and the results may be influenced by subjective factors. Furthermore, the absence of long-term evaluation regarding the impact of narrative medicine programs limits the understanding of their potential influence on the development of longitudinal intervention strategies [14]. The role of social media in health communication is increasingly significant, and nursing students demonstrate a high level of engagement with social media platforms [14]. Therefore, the research on health communication courses exhibits good innovation and feasibility. Sun et al. carried out randomized controlled experiments to conduct an intervention using health communication courses, while the blinding experimental design was not used, leading to the reduced reliability of the research results. The concept of penguin style training is derived from the cooperative breeding behavior of emperor penguins in harsh and densely populated environments, serving as a team-oriented pedagogical approach [16]. This training method is particularly applicable to departments and groups characterized by close-knit teamwork, such as operating rooms [16]. In the study conducted by Dong et al., although the efficacy of the penguin style breeding method in improving nursing professionalism has been verified, the limited sample size may have slightly compromised the statistical significance. In addition, quasi-experimental research was employed in their study. The research design of the quasi-experimental study is not rigorous, and there are different degrees of deviations from the allocation of research objects, the implementation of intervention measures, and the measurement of outcome indicators, which reduce the accuracy of the research results. Previous studies have shown that randomized controlled trials are the highest level of evidence for evaluating the effectiveness of interventions [42]. Therefore, future interventional studies should prioritize standard randomized controlled trials in order to enhance the professionalism of nursing. However, it is worth noting that certain intervention plans lack comprehensive design principles, reference models, or theoretical foundations. Due to the complexity of the concept and the diversity of influential factors, the development of the plans should involve multidisciplinary teams and formulate multilevel, scientifically effective intervention plans [43]. In view of this, the formulation of intervention plans should be based on mature and comprehensive models as theoretical guidance, aiming to develop scientifically rigorous multidisciplinary intervention plans. In the future, mixed research methods should also be used to improve the investigation of influential factors that promote or inhibit nursing professionalism and their underlying mechanisms.

## 5. Conclusion

Currently, the connotation of nursing professionalism needs to be enriched, which will facilitate further exploration of the level of nursing professionalism in the future. Although there are various assessment tools for nursing professionalism, their reliability and validity still need to be verified, and specific assessment tools applicable to different cultural backgrounds are urgently required. The research methods of influential factors related to nursing professionalism should be diversified, while further enhancements are needed for interventional research designs. The following research should prioritize the exploration and construction of nursing professionalism's connotation, by drawing upon well-established theoretical models from various countries for the development of assessment tools. In addition, employing mixed research methods can facilitate the investigation of influential factors associated with nursing professionalism, and diverse intervention measures can be implemented to enhance professionalism levels and indirectly improve the quality of nursing services.

## 6. Implications for Nursing Management

The study of nursing professionalism provides an in-depth understanding of nursing work and offers guidance for nursing managers to support the development of nursing staff and improve their job satisfaction. Nursing professionalism constitutes a fundamental aspect of nursing practice, contributing to the improvement of the quality of nursing care, enhancement of cooperation between medical teams, and growth of reputation of medical institutions [30]. To ensure the safe and efficient working of nurses, nursing managers should provide a conducive working environment with necessary facilities and equipment, enhance their professional education and training, as well as strengthen their professional awareness and sense of responsibility. In addition, incentive mechanisms should be established to reward and commend outstanding nurses, thereby fostering their motivation to actively showcase their professional abilities and uphold professionalism. Only through continuous enhancement of their competence can nurses deliver high-quality nursing services to patients and achieve the sustainable development of the nursing profession.

## Figures and Tables

**Table 1:** Characteristics of the included studies (*n* = 15).

Author (year)	Country	Subjects	Study design	Implementation level (score)	Evaluation instruments	Influencing factors	Intervention strategies (intervention duration)
Zhang et al. [5]	China	Nursing students	Cross-sectional	Medium (69.83 ± 9.01)	Hall's professionalism inventory (Chinese version)	Gender; reasons for applying; willingness and recognition of nursing; role model	— (—)

Xue et al. [14]	China	Nursing students	Randomized controlled trials	—	Hall's professionalism inventory (Chinese version)	—	Control group: diary writing; Experimental group: narrative medicine and narrative writing theory courses (12 months)

Sun et al. [15]	China	Nursing students	Randomized controlled trials	—	Hall's professionalism inventory (Chinese version)	—	Control group: conventional nursing education, including courses in nursing humanities, nursing psychology, nurse skills training and basic nurses Courses; Experimental group: an additional health communication education course on the basis of conventional care education (3 months)

Dong and Shao [16]	China	Nurses	Quasi-experimental	—	Hall's professionalism inventory (Chinese version)	—	Control group: newly enrolled nurses receive one-on-one class-based training once a week, which includes intensive instruction on hospital systems, theoretical knowledge of the operating room, and operational skills; Experimental group: standardized training in penguin culture (including a professional tutor responsible for imparting knowledge to newly enrolled nurses, a mental mentor responsible for cultivating the desired attitude among these nurses, a training partner responsible for skills training, and a supervisor responsible for the supervision of the curriculum development and teaching methodologies) (12 months)

Ma et al. [17]	China	Nurses	Cross-sectional	Low (—)	Self-designed questionnaire	Personal factors; hospital factors; social factors	— (—)

Yu et al. [18]	China	Nurses	Cross-sectional	Low (83.41 ± 8.75)	Hall's professionalism inventory (Chinese version)	Gender; whether the first choice of college entrance examination is nursing major; first education; employment form; receipt of nursing-related rewards for the last 3 years	— (—)

Gyung [19]	Korea	Nursing students	Cross-sectional	—	Nursing professionalism scale	Critical thinking; leadership	— (—)

Kurucová et al. [20]	Czechoslovakia	Nurses	Cross-sectional	Medium (136.74 ± 32.66)	Nurses' professionalism inventory	Professional ranks and titles; education background	— (—)

Pareek and Batra [21]	India	Nurses	Qualitative	—	Focus interview group	Personal factors; organizational factors; environmental factors	— (—)

Wu et al. [22]	China	Nursing students	Cross-sectional	Medium (67.76 ± 6.40)	Hall's professionalism inventory (Chinese version)	Humanitarian care Capacity; social support	— (—)

Zhao et al. [23]	China	Nurses	Mixed	—	Nurse professionalism questionnaire	Gender; professional ranks and titles; education background; work years	— (—)

Ichikaw et al. [24]	Japan	Nurses	Mixed	—	Nurses' professionalism inventory	Beliefs and attitudes on the basis of professional behavior in nurses	— (—)

Tanaka et al. [25]	Japan	Nurses	Cross-sectional	Low (6.74 ± 3.89)	Behavioral inventory for professionalism in nursing	Education background; work years; whether the current nursing manager	— (—)

Tanaka et al. [26]	Japan	Nurses	Cross-sectional	Low (9.19 ± 3.89)	Behavioral inventory for professionalism in nursing	Nursing experience; education preparation; the current position as a nurse administrator	— (—)

Kim and Park [27]	Korea	Nurses	Cross-sectional	Medium (96.64 ± 13.48)	Korean-nursing professional value	Self-efficacy; job embeddedness	— (—)

*Note*. — means “none.”

**Table 2:** Assessment tools of nursing professionalism (*n* = 7).

Evaluation instruments	Country	Author (year)	Scoring system	Dimension number/dimension name	Number of items	Reliability and validity
Hall's professionalism inventory (Chinese version)	China	Wu [28]	Likert 5	6/participation in group organization; public service concept; professional autonomy; self-discipline; sense of professional mission; work satisfaction	25	Cronbach's *α* 0.750; test-retest reliability 0.840; —

Behavioral inventory for professionalism in nursing	American	Miler [3]	—	9/educational preparation; autonomy; theory; code of ethics; community service; competence; publication; research; professional organizations	48	Cronbach's *α* 0.760; —

Nurses' professionalism inventory	Japan	Ichikawa [24]	Likert 6	5/accountability; self-improvement; professional attitudes; nursing professional development; professional members	28	Cronbach's *α* 0.950; test-retest reliability 0.940; —

Self-designed questionnaire	China	Ma [6]	—	4/professional ethics; professional awareness; professional skills; personality characteristics	40	Cronbach's *α* 0.778; —

Nurse professionalism scale	Korea	Gyung [19]	Likert 5	5/professional self-concept; social awareness; nursing professionalism; nursing role and nursing originality	18	Cronbach's *α* 0.870; —

Nurse professionalism questionnaire	China	Zhao [23]	Likert 5	5/unity and cooperation spirit; professional identity; professional development of independent consciousness; spirit of self-determination; dedication	19	Cronbach's *α* 0.737; content validity 0.859

Korean-nursing professional value	Korea	Yeun et al. [29]	Likert 5	5/professional self-concept; social recognition; nursing expertise; nursing competence; nursing autonomy	29	Cronbach's *α* 0.920 —

*Note*. — means not provided.

## Data Availability

Data supporting the findings of this study are available upon reasonable request from the corresponding author.

## Supplementary Materials

Supplementary Materials S1: document retrieval strategies in various databases. This file includes the detailed search strategies for each database.
