# Supplementary material for: Nursing Professionalism: A Scoping Review of Implementation Level, Evaluation Instruments, Influential Factors, and Intervention Strategies
Source: J Nurs Manag. 2024 Aug 20;2024:7272296. doi: 10.1155/2024/7272296 (PMC11918933; doi:10.1155/2024/7272296)
Supplement: Supplementary Materials — S1: document retrieval strategies in various databases. This file includes the detailed search strategies for each database. [file 7272296.f1.docx]

**Supplemental files1: Search strategy**

**PubMed：**

#1 ((((((Professionalism[MeSH Terms]) OR (Professionalism[Title/Abstract])) OR (Professional spirit[Title/Abstract])) OR (Medical professionalism[Title/Abstract])) OR (Professionalism education[Title/Abstract])) OR (Occupational spirit[Title/Abstract])) OR (Vocational spirit[Title/Abstract])

#2 (((((((Nurses[MeSH Terms]) OR (Nurses[Title/Abstract])) OR (nursing[MeSH Terms])) OR (nursing[Title/Abstract])) OR (Nursing students[Title/Abstract])) OR (Nursing personnel[Title/Abstract])) OR (Registered nurses[Title/Abstract])) OR (Pupil nurses[Title/Abstract])

#3 Date-piblication 2023/7/13

#4 #1 AND #2 AND #3

**Web of Science**

(TS=(“professional spirit” OR “Professionalism” OR “medical professionalism” OR “professionalism education” OR “occupational spirit” OR “vocational spirit”) AND TS=("Nurse" OR "Nursing" OR "nursing student" OR "nursing personnel" OR "registered nurse" OR “pupil nurse”) and LA=(English))

data base= WOS, BCI, KJD, MEDLINE, RSCI, SCIELO

Time span = 1950-2023.7.13

**the Cochrane Library:**

“professional spirit” or “Professionalism” or “medical professionalism” or “professionalism education” or “occupational spirit” or “vocational spirit” in Title Abstract Keyword AND "Nurse" or "Nursing" or "nursing student" or "nursing personnel" or "registered nurse" or “pupil nurse” in Title Abstract Keyword - with Cochrane Library publication date to July 13, 2023

**EMBASE:**

('professionalism'/exp OR professionalism:ti OR 'professional spirit':ti OR 'medical professionalism':ti OR 'professionalism education':ti OR 'occupational spirit':ti OR 'vocational spirit':ti) AND ('nurse'/exp OR 'nursing'/exp OR nurse:ti OR nursing:ti OR 'nursing student':ti OR 'nursing personnel':ti OR 'registered nurse':ti OR 'pupil nurse':ti) AND [1966-2023]/py

Time span = <1966-2023.7.13

**CNKI:**

SU=(*Zhiyejingshen* + *Zhuanyejingshen*) * (*Husheng* + *Hushi* + *Huli*)

Time limitation: from database building to 2023/7/13

**Wanfang:**

Title/Abstract: (“*Zhiyejingshen*” OR “*Zhuanyejingshen*”) AND Title/Abstract: (“*Huli*” OR “*Hushi*” OR “*Husheng*”)

Time limitation: from database building to 2023/7/13

**VIP:**

(U=“*Zhiyejingshen*” OR “*Zhuanyejingshen*”) AND (U=“*Huli*” OR “*Hushi*” OR “*Husheng*”)

Time limitation: from database building to 2023/7/13
